# Supplementary material for: Hyperbranched Poly(β-amino ester)s (HPAEs) Structure Optimisation for Enhanced Gene Delivery: Non-Ideal Termination Elimination
Source: Nanomaterials (Basel). 2022 Nov 4;12(21):3892. doi: 10.3390/nano12213892 (PMC9656648; doi:10.3390/nano12213892)
Supplement: Supplementary file 1 [file nanomaterials-12-03892-s001.zip › nanomaterials-1987911-SI.pdf]

SUPPORTING INFORMATION

Hyperbranched Poly(β-amino ester)s (HPAEs) Structure Optimisation for Enhanced Gene Delivery: Non-Ideal Termination Elimination

Yinghao Li [1,†], Zhonglei He [1,2,†], Jing Lyu [1], Xianqing Wang [1], Bei Qiu [1], Irene Lara-Sáez [1], Jing Zhang [3], Ming Zeng [4], Qian Xu [1], Sigen A [1], James F. Curtin [2,5,*], and Wenxin Wang [1,*]

1 Charles Institute of Dermatology, School of Medicine, University College Dublin, D04 V1W8, Dublin, Ireland
2 BioPlasma Research Group, School of Food Science and Environmental Health, Technological University Dublin, D07 H6K8, Dublin, Ireland
3 State Key Laboratory of Materials-Oriented Chemical Engineering, College of Chemical Engineering, Nanjing Tech University, 30 Puzhu South Road, Nanjing 211816, China
4 Department of Dermatology, The First Affiliated Hospital of Jinan University, Guangzhou Overseas Chinese Hospital, Guangzhou 510630, China
5 Faculty of Engineering and Built Environment, Technological University Dublin, D07 H6K8, Dublin, Ireland
* Correspondence: james.curtin@tudublin.ie (J.C.); wenxin.wang@ucd.ie (W.W.)
† These authors contributed equally to this work.

Supplementary Figures

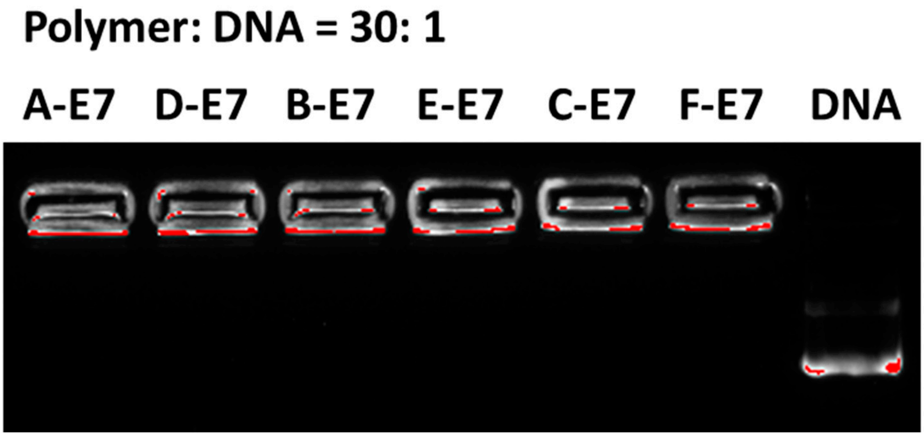

Figure S1. DNA condensation capacity of HPAEs at a w/w ratio of 30:1.

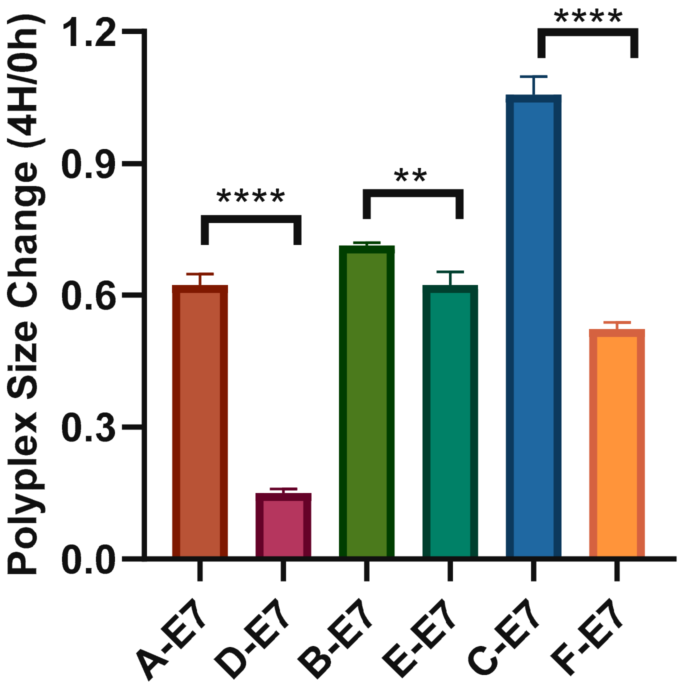

Figure S2. Polyplex stability in serum measure evaluated by the relative size change post 4h incubation at 37ºC of HPAEs at a w/w ratio of 30:1.

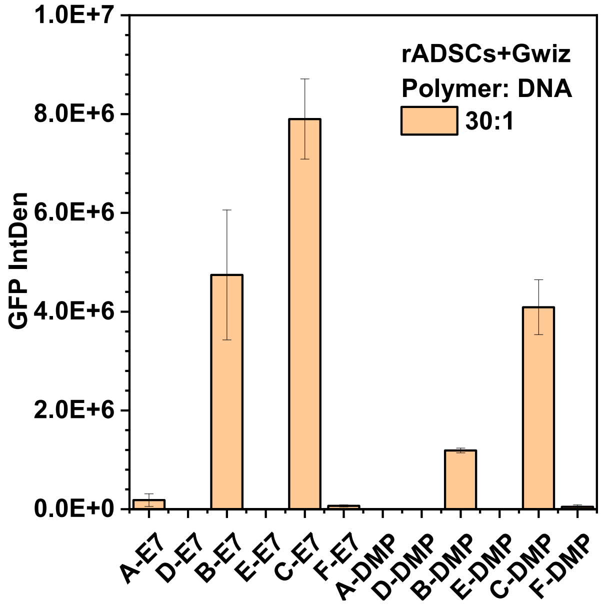

Figure S3. GFP expression of rADSCs cells post-transfection by different HPAE-based polyplexes at the ratio of 30:1 (polymer/ DNA w/w).

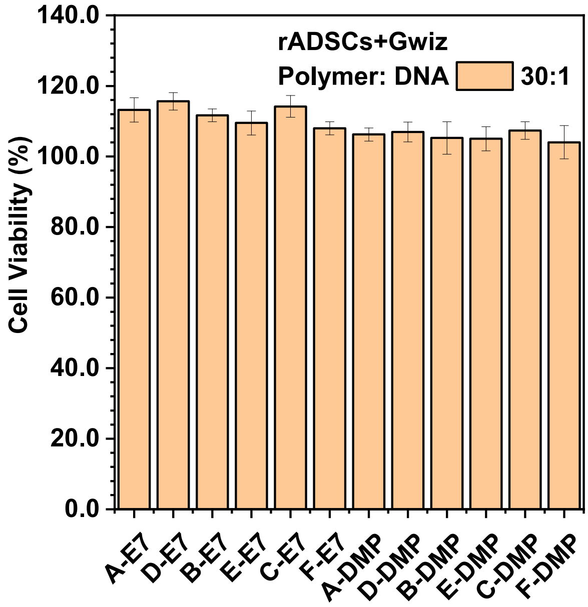

Figure S4. Cell viability of rADSCs cells post-transfection by different HPAE-based polyplexes at the ratio of 30:1 (polymer/ DNA w/w).
